# Lead-free relaxor-ferroelectric thin films for energy harvesting from low-grade waste-heat

**DOI:** 10.1038/s41598-020-80480-1

**Published:** 2021-01-08

**Authors:** Amrit P. Sharma, Makhes K. Behera, Dhiren K. Pradhan, Sangram K. Pradhan, Carl E. Bonner, Messaoud Bahoura

**Affiliations:** 1 Center for Materials Research, Norfolk State University, 700 Park Ave, Norfolk, VA 23504 USA; 2 Geophysical Laboratory, Carnegie Institution for Science, Washington, DC 20015 USA; 3 Engineering Department, Norfolk State University, 700 Park Ave, Norfolk, VA 23504 USA

**Keywords:** Devices for energy harvesting, Electronic devices

## Abstract

One of the ways to mitigate the world energy crisis is to harvest clean and green energy from waste-heat, which is abundant, ubiquitous, and free. Energy harvesting of this waste-heat is one of the most encouraging methods to capture freely accessible electrical energy. Ferroelectric materials can be used to harvest energy for low power electronic devices, as they exhibit switchable polarization, excellent piezoelectric and pyroelectric properties. The most important characteristic of ferroelectric materials, in the context of energy harvesting, is their ability to generate electric power from a time-dependent temperature change. In this work, we grew highly c-axis oriented heterostructures of BaZr_0.2_Ti_0.8_O_3_ (barium zirconium titanate, BZT)/Ba_0.7_Ca_0.3_TiO_3_ (barium calcium titanate, BCT) on SrRuO_3_ (strontium ruthenate, SRO) and deposited on SrTiO_3_ (strontium titanate, STO) single crystalline substrate using pulsed laser deposition (PLD) technique. We investigated the structural, electrical, dielectric, and pyroelectric properties of the above-mentioned fabricated heterostructures. The wide range of θ–2θ X-ray diffraction (XRD) patterns only shows (*00l*) reflection peaks of heterostructures and the substrate which confirmed that the films are highly c-axis oriented. We are also capable to convert the low-grade waste-heat into electrical energy by measuring various temperature-dependent ferroelectric hysteresis loops of our nanostructure films via pyroelectric Ericsson cycles and the structures show an energy conversion density ~ 10,970 kJ/m^3^ per cycle. These devices exhibit a large pyroelectric current density of ~ 25 mA/m^2^ with 11.8 °C of temperature fluctuation and the corresponding pyroelectric coefficient of 3425 μC/m^2^K. Our research findings suggest that these lead-free relaxor-ferroelectric heterostructures might be the potential candidates to harvest electrical energy from waste low-grade thermal energy.

## Introduction

The increase of energy demand over the years and its cost coupled with different environmental issues, such as greenhouse effect, global warming, air pollution, and acid rain^1,2^, drive the world to go for cleaner, more reliable and renewable energy resources (i.e., wind, solar, heat, stress, vibration, and magnetic field). In the United States, around 68% of the primary energy generated is wasted as heat each year^3^. The waste-heat is ubiquitous, abundant, and freely available^4^ making energy-harvesting a cost-effective, maintenance-free, and self-sustainable, hence they are suitable for supplying power in remote areas. Waste-heat is classified into three types, depending on the temperature range^5^: high-grade (≥ 649 °C), medium grade (232—649 °C), and low-grade (≤ 232 °C). Usually medium and high-grade heat can be easily recovered when compared to low-grade waste heat. However, a substantial amount ~ 60% of the wasted heat is low-grade, which is difficult to scavenge efficiently^5^.

Considerable efforts are currently underway to utilize the low-grade waste-heat with improved efficiency and reduced carbon emission using pyroelectric (ferroelectric) materials which require periodic temperature fluctuation.

Ferroelectric materials demonstrate a spontaneous and switchable polarization, which are strongly electric field and temperature dependent. The change of polarization with the variation of temperature is called the pyroelectric effect (PEE). This occurs in ceramics, single crystals, thin films, and polymers with an acentric crystal structures possessing a unique axis of rotational symmetry, giving the structure an inherent spontaneous polarization^6^.

The pyroelectric performance of a material is determined by its pyroelectric coefficient (π), which is the rate of change of polarization with temperature and can be computed by probing the pyroelectric current,

1 $${i}_{p }=\pi A\left(\frac{dT}{dt}\right)$$

where *A* is the area of the electrode and dT/dt is the rate of change of the material’s temperature with respect to time. Many techniques have been reported to measure the pyroelectric properties of large samples^7,8^. However, it is more challenging to estimate π in smaller samples because of poor accuracy in temperature measurement, non-uniform heating, and thermally induced current produced when the trapped electrons are released^9,10^. Only a few techniques, such as, hot plate heating, micro-fabricated resistive heater-based experiments^11^ and modulated laser-based measurements^12^ are reported to estimate π for small-sized samples.

The power density of different thermal energy harvesting techniques is published in the literature. The thermoelectric generators show very high-power density (~ 3.0 kW/cm^3^) when the difference in temperature is > 600 °C. But their power density significantly decreases with the decrease of temperature difference. Nguyen et al.^13^ has reported a power density of 10.7 mW/cm^3^ at a temperature difference of 14.8 °C in P(VDF-TrFE) pyroelectric material whereas Sato et al.^14^ observed power density of ~ 2.0 mW/cm^3^ on thermoelectric material (shape memory alloy) at similar temperature difference. Therefore, for the small temperature difference (ΔT < 100 °C), pyroelectric materials are one of the most capable of waste-heat scavenging exhibiting an appreciable power density^4,15^.

To this date, only a few materials were studied to recover waste heat resulting in some exciting results. Recently, Pandya et al*.*^16^ studied the conversion of pyroelectric energy from low-grade waste-heat, which exploits both temperature and field-dependent polarization, on lead-based 0.68Pb(Mg_1/3_Nb_2/3_)O_3_-0.32PbTiO_3_ relaxor-ferroelectric thin films. Similarly, Bhatia et al.^11^ demonstrated three frequency domain thermal measurement (a hot plate, microfabricated heater, and modulated laser) techniques and compared their corresponding pyroelectric currents based on PZT epitaxial film from 0.02 Hz to 1.3 MHz. To date, most of the studies have been focused on lead-based ferroelectric materials and reported their energy conversion density. Even though the lead-based ferroelectric materials exhibit large polarization with excellent pyroelectric as well as piezoelectric coefficients, their applications are limited because of their toxicity and environmental concerns.

Regarding the lead-free ferroelectric materials, Bhatia et al.^17^ systematically studied a BaTiO_3_ thin film and presented detailed studies, using the pyroelectric Ericsson cycle, to optimize the conversion of thermal energy into electrical energy. While BaTiO_3_ is one of the most popular lead-free ferroelectric materials, its ferroelectric, dielectric, piezoelectric, and pyroelectric properties are lower than the lead-based ferroelectrics. Previous research showed the enhancement of these properties by suitable site modification at both sites in BTO solid solutions BaZr_0.2_Ti_0.8_O_3_ (BZT), Ba_0.7_Ca_0.3_TiO_3_ (BCT), and BaZr_0.2_Ti_0.8_O_3_–Ba_0.7_Ca_0.3_TiO_3_–(BZT-BCT) for different applications in multifunctional devices^18–20^. Recently, Patel et al*.*^15^ reported a high thermomechanical energy conversion density on lead-free BZT-BCT bulk ceramics. However, there is a lack of studies on lead-free relaxor-ferroelectric multilayer structure for thermal energy harvesting.

In this study, we present our efforts to improve the performance of relaxor-ferroelectric thin-film heterostructures (BCT/BZT) for low-grade waste-heat conversion. Our previous study^18,21^ demonstrated that relaxor-ferroelectric heterostructures showed larger energy density, owing to their high breakdown strength, large polarization, and low remnant polarization. Nevertheless, to the best of our knowledge, this study is the first attempt to recover low-grade waste-heat using lead-free relaxor-ferroelectric based on multilayer structures. We fabricated the devices with capacitive structures 200 nm thick of BCT/BZT bilayer thin films inserted between Pt (platinum) top electrode and SRO bottom electrode. The device enabled the investigation of thermal harvesting in the heterostructure in response to a periodic temperature fluctuation.

## Methods

### Thin film fabrication

We prepared the BCT and BZT ceramic targets by a high-temperature solid-state reaction method following the procedures reported in our previous work^18^. Those targets are sintered at a high temperature, 1400 °C, for 6 h and utilized them for the growth of the BZT/BCT thin film structures by PLD technique, which uses a KrF excimer laser with energy density ~ 2 J/cm^2^ and wavelength λ ~ 248 nm. First, we grew ~ 60 nm of SRO buffer layer on STO (100) single crystal substrate. Then ~ 200 nm of BZT/BCT multilayers are grown on SRO deposited STO substrate. The optimized deposition parameters of BCT, BZT, and SRO thin films are summarized in Table 1. Finally, the as-grown samples are annealed at 800 °C under an oxygen environment of 300 Torr for 30 min followed by cooling to room temperature.

**Table 1:** Optimized deposition parameters for the epitaxial growth of different materials.

Material	Substrate temperature (°C)	Oxygen pressure (mTorr)	Annealing temperature and time	Laser repetition (Hz)
BCT	800	100	800 °C × 30 min	7
BZT	800	100	800 °C × 30 min	7
SRO	800	20	800 °C × 30 min	7

**Figure 1:**
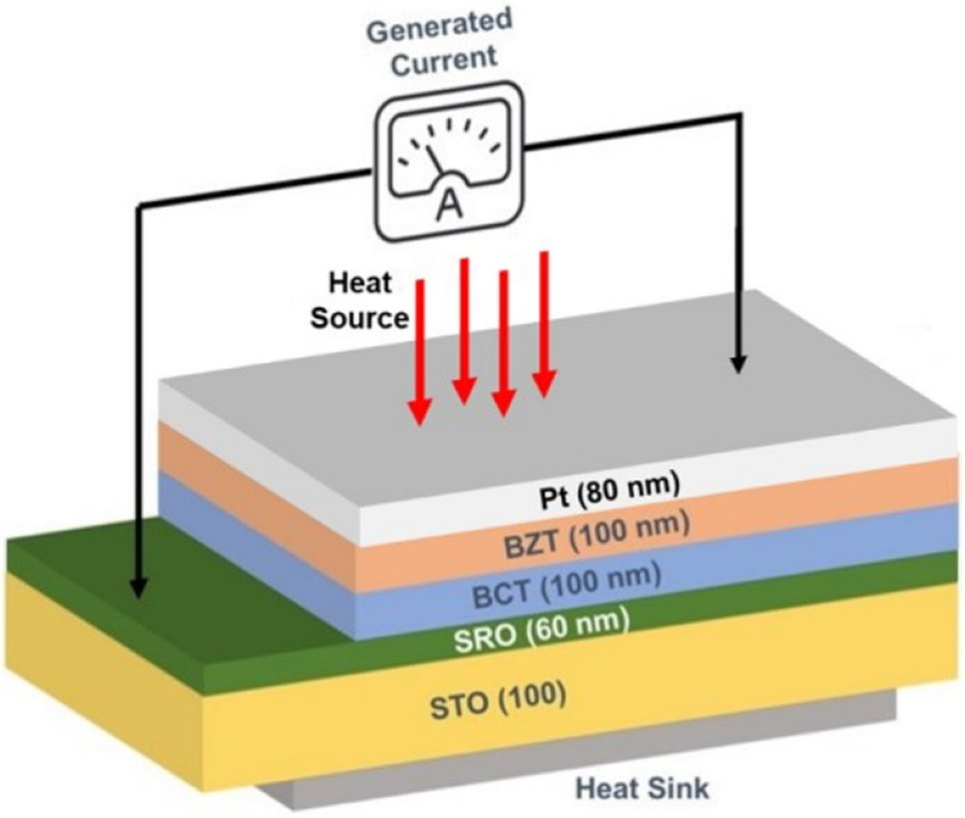
Schematic of the capacitive structure of a pyroelectric device.

Thin-film of platinum (Pt) with dimension (6 mm × 4 mm) and thickness ~ 80 nm is deposited as a top electrode on the heterostructures via electron beam evaporation technique by means of a shadow mask to form the capacitor with metal-ferroelectric-metal (MFM) configuration, as shown in Fig. 1. This capacitor structure efficiently harvests the pyroelectric current produced by heating the BCT/BZT multilayer structures with a periodically modulated laser.

### Characterization

The phase purity, atomic spacing, and crystal structure of the heterostructures are examined using an X-ray diffractometer (Rigaku D/Max-2200 TB) in a wide range of θ–2θ (20°–80°) scan at 40 mA and 40 kV with CuK_α_ radiation (λ = 1.5405 Å) at a speed of 0.125° per minute at ambient temperature. The thickness of the heterostructures is measured using a profilometer (DektakXT, Bruker Corporation) whereas its surface morphology is studied using Bruker’s Dimension Icon atomic force microscopy (AFM).

The electrical and dielectric measurements are carried out with a Keithley 4200 semiconductor characterization system and an HP4294A impedance analyzer. The ferroelectric hysteresis (P-E) loops, at various temperatures, are recorded using a hysteresis loop tracer (Radiant Technologies Inc.).

### Pyroelectric measurement

**Figure 2:**
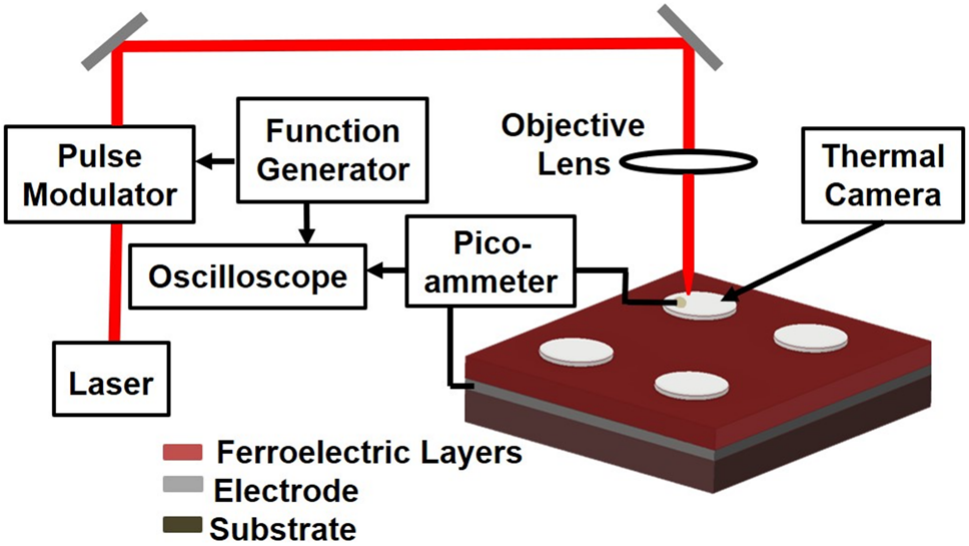
Schematic description of the pyroelectric measurement.

Figure 2 describes a schematic diagram of the indigenously designed pyroelectric measurement set up. The pyroelectric response of the sample is observed using a periodic heating/cooling method, with an adjustable duty cycle. A cw-laser diode operating at a wavelength of 850 nm is modulated in a wide frequency range (10 mHz–10 kHz) by a function generator and the modulated output of the diode is concentrated on the platinum electrode layer with a 5X objective lens. The 1/e^2^ intensity radius of the perfectly aligned laser spot is ~ 100 µm. The intensity modulation of the laser power is absorbed by the BZT/BCT layer to produce an oscillating temperature within the ferroelectric film with the same frequency and negligible phase shift. Care was taken to avoid direct illumination of the soldered electrode used to collect current. The direct illumination of the soldered electrode resulted in a waveform with a single polarity. This was the case for the higher repetition rates and laser intensities. This single polarity waveform can be attributed to the thermionic emission of the soldered electrode. The other possibility might be due to the current generated by thermomechanical-induced strains in the pyroelectric layer.

To produce the symmetric-temperature fluctuation of 11.8 °C, the average laser power and frequency are chosen to be 150 mW and 50 mHz, respectively. The actual temperature fluctuation on the sample is measured using a thermal camera (FLIR A Series) set to measure at 15 frames per second. The observed pyroelectric current is measured from the top platinum and bottom SRO electrodes using a Pico-ammeter (Keithley 6485) whose signal is, subsequently, fed to a digital oscilloscope (Tektronix TDS 740) for signal averaging. The average of a minimum of 100 traces are collected.

## Results and discussion

### Structural studies

**Figure 3:**
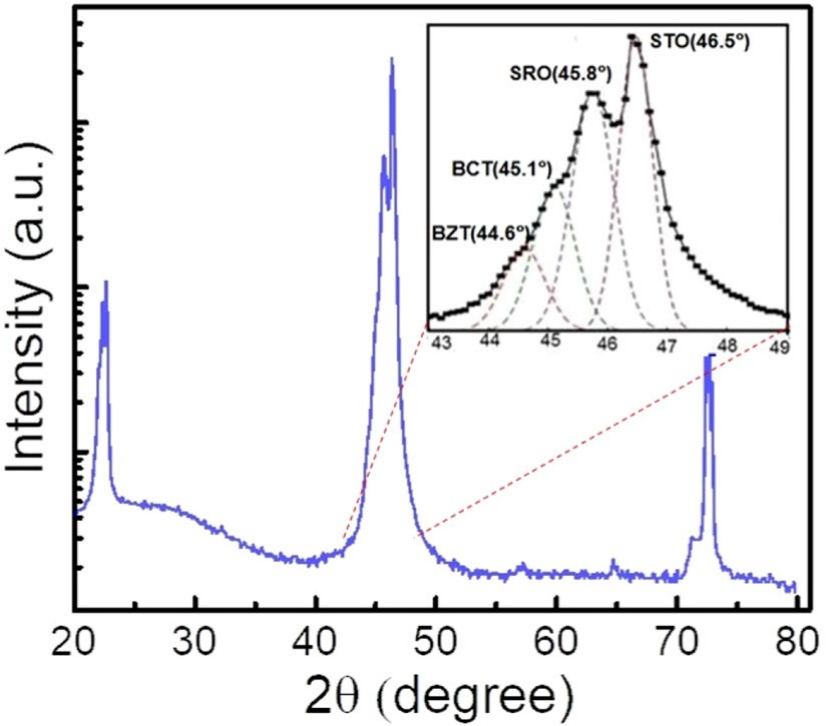
XRD patterns of the heterostructures BZT/BCT on SRO deposited STO (100) substrate recorded at room temperature. Inset displays the enlarged version of (002) peaks of BZT, BCT, SRO, and STO.

The crystal structure, phase purity and orientation of BZT/BCT heterostructures grown on SRO deposited STO substrate were studied using the X-ray diffraction technique. Wide-angle θ–2θ scans (20°–80°) of a 200 nm thick BZT/BCT on 60 nm SRO/STO (100) heterostructures recorded at room temperature are shown in Fig. 3. No extra peaks were seen except the reflection peaks from the substrate and BZT/BCT heterostructures, which confirmed the heterostructure is (00* l*)-oriented, single-phase, and of good quality with highly c-axis oriented without any secondary phases. The inset exhibits the observed diffraction peak positions of (002) peaks of BZT ~ 44.6°, BCT ~ 45.1°, SRO ~ 45.8° and STO ~ 46.5°, obtained after fitting the peak found between 43° and 49° to a Gaussian profile. The true lattice constant of BCT and BZT computed using Nelson–Riley fit are found to be ~ 3.957 Å and ~ 4.008 Å, respectively.

### Morphological characterization

**Figure 4:**
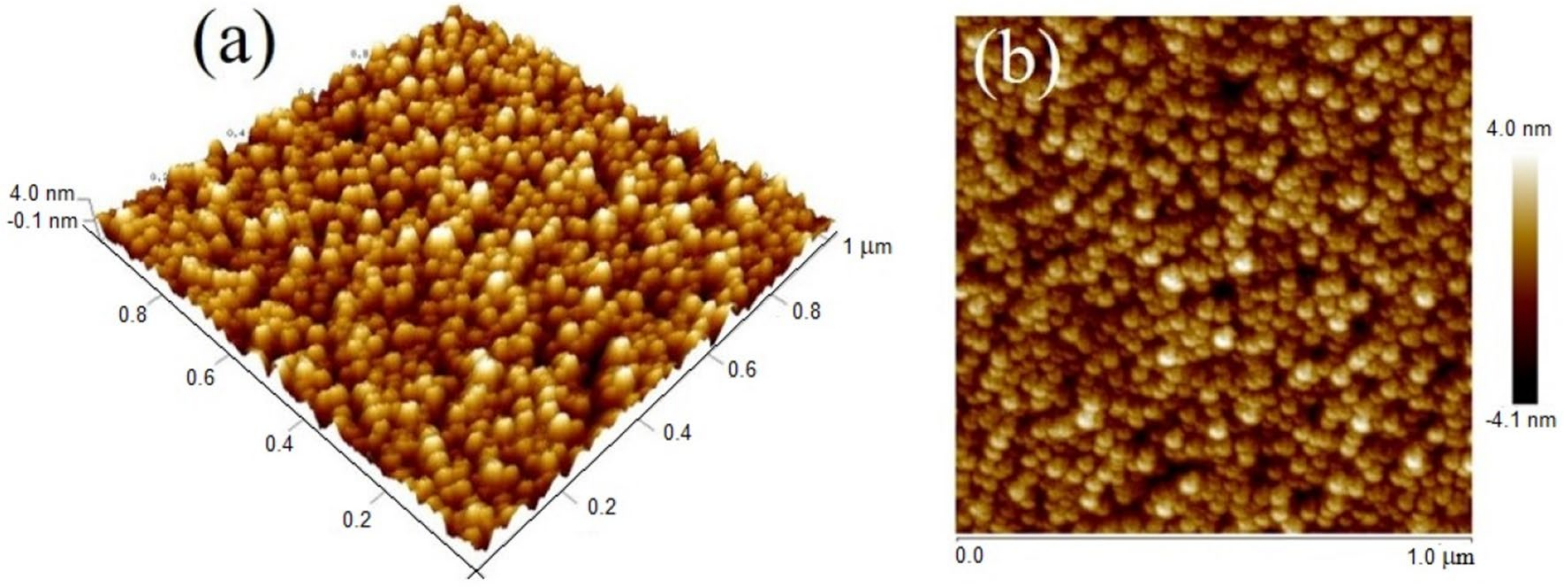
AFM images of the heterostructures BZT/BCT: (**a**) 3D and (**b**) 2D.

The surface morphology of BZT/BCT heterostructures, captured using atomic force microscopy (AFM) operated in tapping mode, are shown in Fig. 4a,b. The images are scanned for a size of 1 × 1 μm^2^ at multiple locations, which exhibits relatively smooth, well-connected grains with an RMS surface roughness (*R*_q_) of ~ 1 nm. The 3D micrograph, in Fig. 4a, shows a good quality film due to uniform deposition as the upper surface of the film exhibiting a layer that is homogeneous and free of cracks and pores/holes. Such a well-connected, dense, and uniform granular microstructure with low surface roughness is required to enhance the dielectric, the ferroelectric, the pyroelectric, and the electrical properties of the film. Figure 4b depicts the 2D micrograph, which demonstrates that the surface is smooth with densely packed grains of uniform size ~ 40 nm.

### I-V characteristic of BZT/ BCT heterostructures

**Figure 5:**
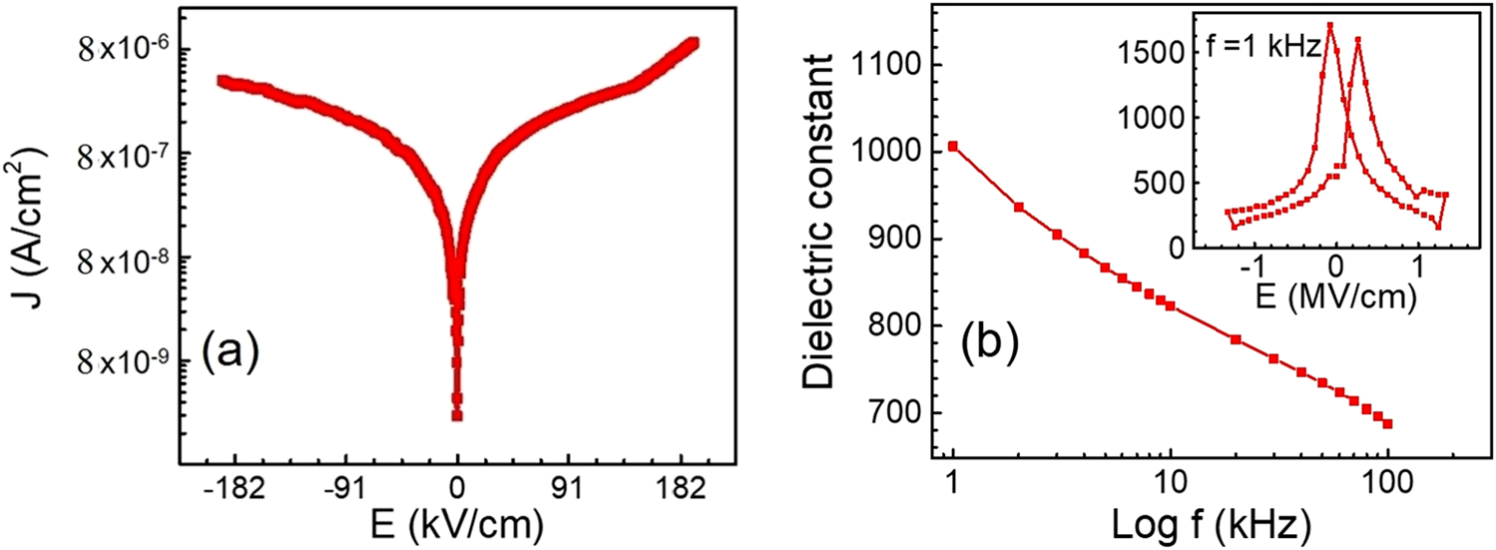
Variation of (**a**) leakage current density with electric field (J–E) curves and (**b**) dielectric constant with frequency and electric field at 1 kHz (inset) of BZT/BCT films.

The current density as a function of electric field (J-E) of BCT/BZT heterostructures in metal-ferroelectric-metal (MFM) architecture at ambient temperature is shown in Fig. 5a. We noticed a low leakage current of < 10^–6^ A/cm^2^ through the film with an applied electric field of 0.1 MV/cm, which is comparable to the literature on similar materials^22,23^. This low leakage current is originated due to the presence of oxygen (100 mTorr) during deposition and the completeness of perovskite phase formation at higher annealing temperature (800 °C) after the deposition^24^. At a higher oxygen environment, the leakage current is reduced by suppressing the defects, such as oxygen vacancies, which reduces the ferroelectricity. There is a sharp increase of current, even at a lower electric field, followed by almost saturation at a higher electric field. At a low electric field, the J-E curve obeys Ohm’s law, at a medium electric field, space-charge induced conduction is dominant whereas, at a higher electric field, Schottky-Frankel conduction mechanism dominates^25,26^.

### Dielectric properties of BZT/BCT heterostructures

Dielectric tunability is one of the important features of the ferroelectrics. The dielectric permittivity of BZT/BCT heterostructures is found to decrease when the frequency increases, Fig. 5b, which indicates a typical characteristic of ferroelectric thin films. The dielectric constant in solids, generally, is due to different types of polarizations such as: (1) space charge/interfacial (2) dipolar/orientational (3) ionic and (4) electronic^27^. At lower frequencies, the dielectric constant is high, which is due to the presence of most of polarization dynamics. The dielectric permittivity of these nanostructures is found to be ~ 1000 at 1 kHz, which is smaller compared to similar materials in the literature^18,28,29^. Such a low dielectric constant is crucial for the improvement of the figure of merit of the pyroelectric device. The variation of dielectric constant with electric field at 1 kHz of the ferroelectric materials is demonstrated in the capacitance–voltage (C–V) characteristics, as shown in Fig. 5b (inset). The polarization modulation, via the variation of the dielectric constant as a function of dc electric field, is related to the domain movement. The higher dielectric response, at a low dc field, can be attributed to the huge increase in polarization due to the reversal of domains. At a higher electric field, the maximum number of domains are aligned along the direction of the electric field, resulting in a significantly reduced dielectric response. Such a typical C–V butterfly characteristic, i.e. non-linear dielectric properties with applied field, demonstrates the ferroelectric nature of the materials. The occurrence of smaller asymmetry in this C–V curve is due to dissimilar materials used for the top (Pt) and bottom (SRO) electrodes.

### Pyroelectric properties of BZT/BCT heterostructures

**Figure 6:**
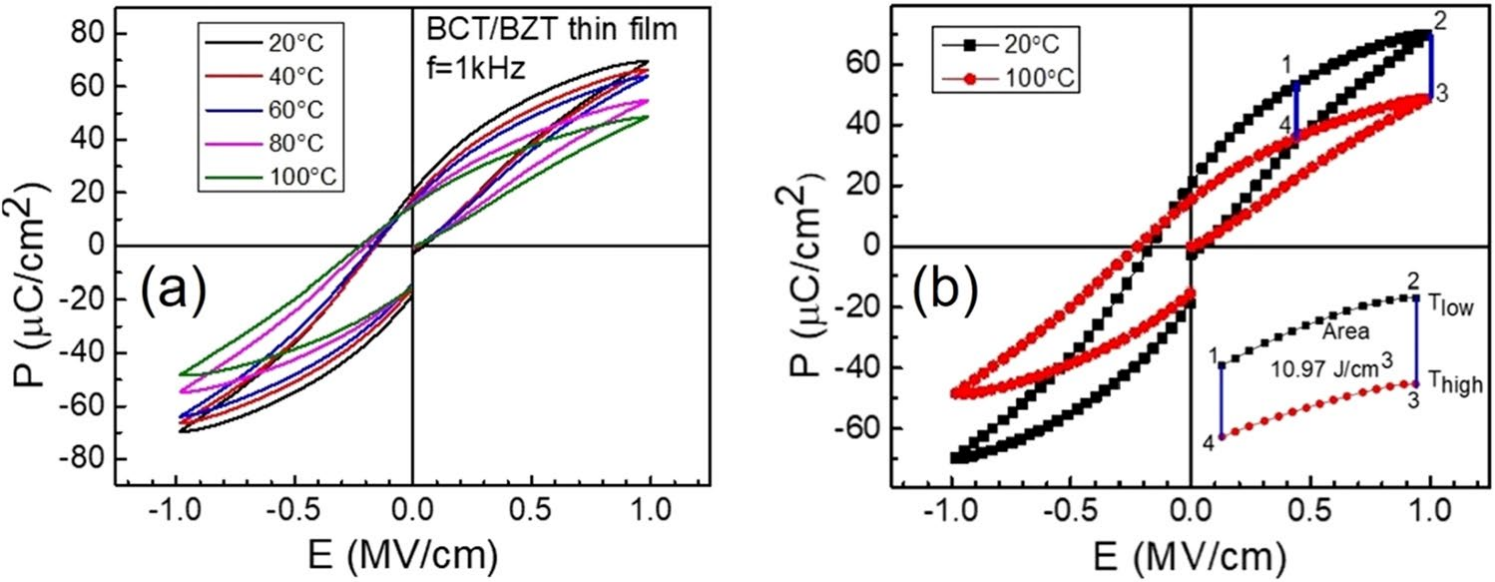
Variation of polarization with electric field (P–E) hysteresis loops of BZT/BCT films at the frequency of 1 kHz at different temperatures (**a**). Sketch of the pyroelectric Ericsson cycle in four-step on a ferroelectric P–E loops at two temperatures (**b**).

As explained in our previous publication^21^, the BCT/BZT heterostructures demonstrated well-saturated and slim ferroelectric hysteresis loops, at ambient temperature in the applied field from 0.7 to 1.7 MV/cm, confirmed the ferroelectric behavior of the heterostructures even at higher applied voltages. Pyroelectric behavior occurs in most of the ferroelectrics as they exhibit large spontaneous and switchable polarization in a wide range of temperature^30^. To estimate the energy conversion density of the thin films of ferroelectrics, we recorded the P-E hysteresis curves of these thin films at different temperatures, Fig. 6a. We found that both the saturation and remnant polarization values decrease monotonically with the increase of temperature^15,29^. The polarization is regulated by the lattice structure of the material and shows a large change when the material undergoes structural transition with the variation of temperature.

A ferroelectric loop is usually recorded in an anticlockwise direction and the area enclosed by the loop indicates the losses by which electrical energy is transformed into thermal energy. However, if the loop is measured in a clockwise direction, it might be possible to generate electrical energy from thermal energy^31,32^. The investigation of pyroelectric energy conversion, with a ferroelectric loop using the Ericsson cycle, is displayed in Fig. 6b and it has the following four steps:

1. Isothermal charging: a high E-field is applied at a constant temperature until it arrives at its saturated polarization where all the dipoles are oriented in the direction of the applied field (represented by step 1–2).
2. Heating at constant E-field: when the material reaches saturated polarization and heats at constant electric field, a large depolarization current is generated due to the change in orientation of the dipoles (represented by step 2–3).
3. Isothermal discharging: once the material achieves thermal equilibrium, the applied E-field is removed slowly, so that all the dipoles are randomly oriented (represented by step 3–4).
4. Cooling at constant E-field: the material at low electric field, removed from the heat source, and then kept at the sink so that all the dipoles restore to their original position giving rise to large polarization current (represented by step 4–1).

The enclosed area 1–2–3–4 by the four steps on a hysteresis loop indicates the energy conversion density (*N*_*D*_) of the thin film, which is computed using the following equation ^33,34^,

2 $${N}_{D}={\oint }_{1234}E dP$$

and found to be 10,970 kJ/m^3^ per cycle. This energy conversion density value is higher than the one found in similar lead-free materials and even higher than some of the lead-based structures as seen in Table 2.

**Table 2:** The pyroelectric energy density harvested per cycle of various recently reported pyroelectric materials.

Materials	T_low_ (K)	T_high__(_K)	E_low_ (kV/cm)	E_high_ (kV/cm)	Pyroelectric energy density harvested (kJ/m^3^ cycle)	References
PbZr_0.53_Ti_0.47_O3/CoFe_2_0_4_ (thin film)	100	300	0	400	47,372	^35^
Hf_0.2_Zr_0.8_O_2_ (thin film)	298	423	0	3260	11,500	^36^
Pb_0.99_Nb_0.02_ (Zr_0.55_Sn_0.40_Ti_0.05_)_0.98_ O_3_ (thin film)	298	498	218	1091	7350	^37^
0.68PbMg_1/3_Nb_2/3_O_3_–0.32PbTiO_3_ (thin film)	303	323	0	600	8000	^38^
0.68PbMg_1/3_Nb_2/3_O_3_–0.32PbTiO_3_ (thin film)	298	388	125	392	1000	^16^
Bi_0.5_Na_0.44_K_0.06_Ti0_3_ (bulk ceramic)	298	393	1	52	1986	^39^
BaZr_0.2_Ti_0.8_–Ba_0.7_Ca_0.3_TiO_3_ (bulk ceramic)	297	369	0	20	149	^15^
BaZr_0.2_Ti_0.8_/Ba_0.7_Ca_0.3_TiO_3_ (multilayer thin film)	293	373	436	985	10,970	This work

There is a great interest in exploring lead-free structures for energy harvesting and recently, it has been found that a maximum energy conversion density of 149 kJ/m^3^ was attained by Patel et al*.*^15^ with a saturated E- field and temperature of 24 °C and 96 °C on a lead-free 50BZT-50BCT bulk system. Similarly, Chauhan et al.^39^ reported a large energy conversion density of 1,986 kJ/m^3^ on a lead-free ferroelectric thin film of Bi_0.5_Na_0.44_K_0.06_TiO_3_ under an operational temperature of 25 °C and 120 °C. The energy conversion density we measured in our lead-free BZT/BCT heterostructures is 10,970 kJ/m^3^ under an operational temperature of 20 °C and 100 °C, which is showing better performance than the other reported data^15,39^.

**Figure 7:**
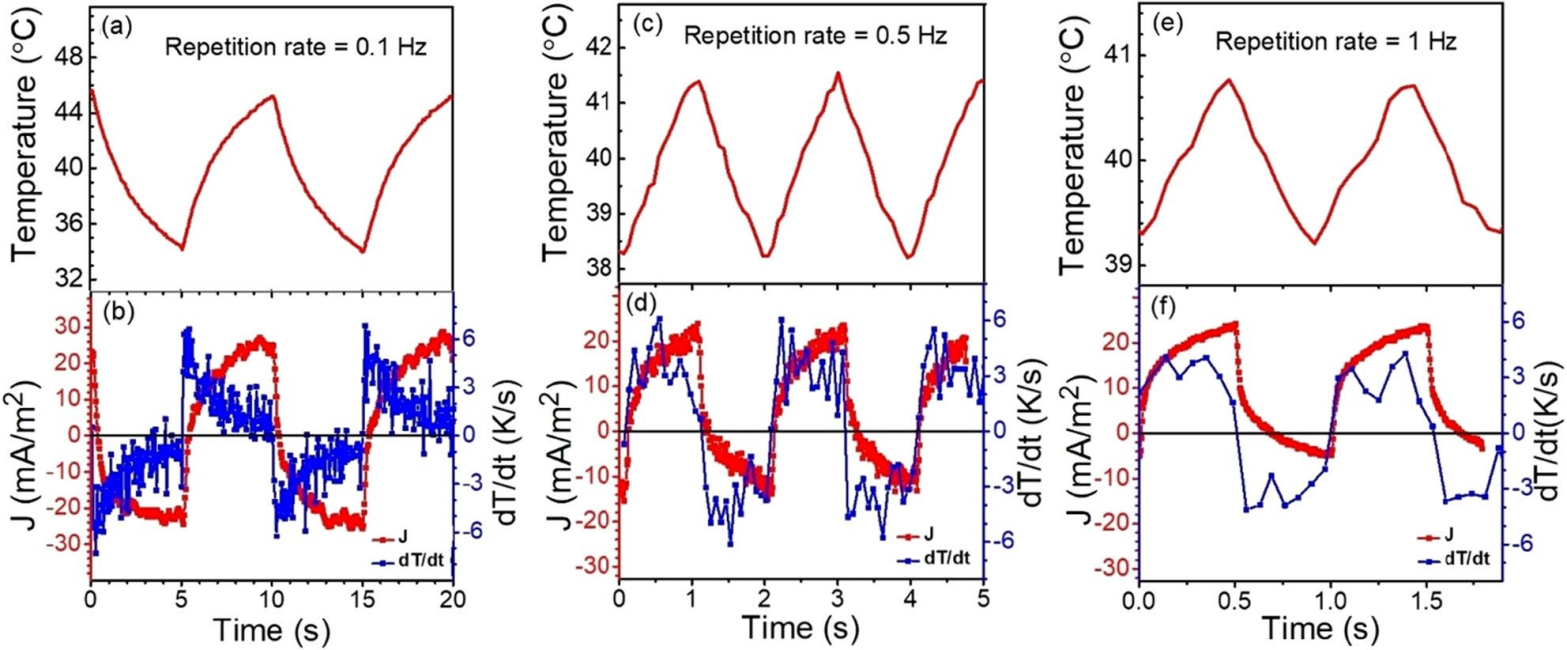
Temporal profile for temperature, its time derivative, and the corresponding pyroelectric current generated (**a**,**b**) for laser repetition rate 0.1 Hz, (**c**,**d**) for 0.5 Hz and (**e**,**f**) for 1 Hz, respectively. At a repetition rate of 0.1 Hz (5 s heating and cooling periods), the observed pyroelectric current is symmetric but at 1 Hz repetition rate (0.5 s heating and cooling periods) the positive current is unchanged and the negative current decreases but is non-zero.

Figure 7 shows the actual temperature fluctuations on the material and the corresponding pyroelectric currents generated from BCT/BZT multilayer structures at different laser illumination repetition rates.

The heating conditions and the observed pyroelectric current maxima and minima are presented in Table 3.

**Table 3:** Temperature range and current density for a range of heating repetition rates.

Heating frequency (Hz)	Temperature difference dT (°C)	Total current density (mA/m^2^)	Positive current density (mA/m^2^)	Negative current density (mA/m^2^)
0.1	11.8	50.8	25.0	25.8
0.2	7.0	35.8	22.3	13.5
0.5	3.4	38.3	23.7	14.6
1.0	1.7	27.9	23.7	4.2

**Figure 8:**
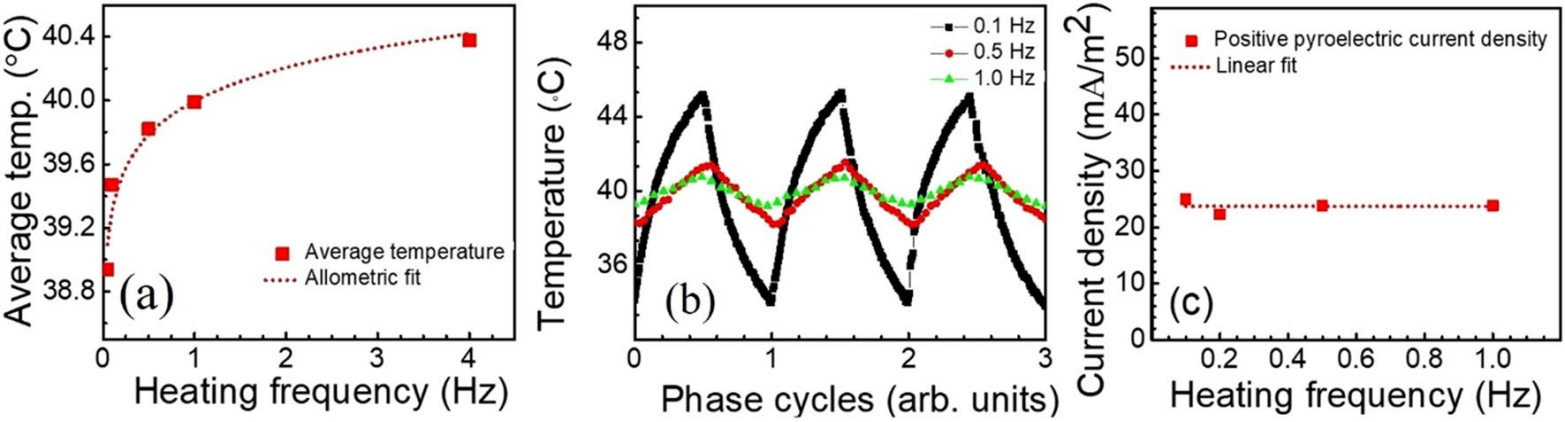
Plot of (**a**) average temperature for different heating frequency, (**b**) the amplitude of temperature change as a function of the phase cycle at heating frequencies, and (**c**) pyroelectric current density versus heating frequency for positive-going current only.

In Table 3, the conditions observed due to laser heating at four different repetition rates are presented with the corresponding temperature ranges observed as well as the current observed during each cycle. As shown in the table, as the repetition rate decreases, with a 50% duty cycle, the period of exposure increases, and the temperature swing experienced by the sample increases as well. Due to this lengthened heating period, the maximum temperature of the sample increases since more heat is delivered to the sample. However, as the heating frequency increases, the average temperature increases slightly since cooling time also decreases. The heating/cooling curves shown in Fig. 8a can be approximated as an exponential rise and fall for 0.1 Hz repetition rate but appear much more linear for faster repetition rates. The temperature range for each repetition rate is presented in Fig. 8b. In the figure, the amplitude is plotted versus the phase of the repetition rate to highlight the amplitude of the oscillation. It is clear from the figure that the amplitude of the heating cycle increases with increasing period. For repetition rates below 0.2 Hz, the cooling period and the cooling time are approaching each other, so that the sample has enough time to reach its unperturbed temperature before the next heating cycle. At repetition rates higher than 0.2 Hz, the sample reaches equilibrium at a higher temperature. This increasing temperature is shown in Fig. 8a. This will be discussed in more detail in a later section of this work.

Figure 7 also contains the waveform of the pyroelectric current generated during each heating and cooling cycle. When the laser repetition rate is 0.1 Hz and a laser incident power of 150 mW and a spot size of ~ 100 microns, the temperature fluctuates with a magnitude of 11.8 ℃ and the corresponding symmetric pyroelectric current is recorded from – 25.8 mA/m^2^ to + 25 mA/m^2^, as shown in Fig. 7a,b. Therefore, at a repetition rate below 0.2 Hz, the pyroelectric waveform is symmetric about the x-axis with approximately the same amplitude in both polarities. As the repetition rate increases above 0.2 Hz, the waveform becomes less symmetric with the amplitude of the negative portion decreasing. When the repetition rate of the laser is increased to 0.5 Hz, a smaller temperature fluctuation of 3.4 °C is measured and the corresponding pyroelectric current is asymmetric with a reduction in the negative current peak value, as shown in Table 3 and also in Fig. 7c,d. With a further increase of laser repetition rate to 1 Hz, the amplitude of the temperature fluctuation is further reduced to 1.7 °C (Fig. 7e,f). At 1 Hz, the negative amplitude of the pyroelectric current is also reduced but is *not* zero. It is worth noting that, both the maximum temperature and positive pyroelectric current only decrease slightly as the incident energy of the laser decreases with the length of the excitation periods. Additionally, the positive current is relatively unchanged with an order of magnitude reduction in the incident energy of the laser excitation.

From the above observations, we can conclude that with increasing laser repetition rates and, thus decreasing heating and cooling periods, the sample cannot cool to its lowest pre-excitation temperature. Therefore, with shorter cooling periods, the temperature to which the sample cools before the next pulse is higher than it would be if left to cool longer. The competition between the heating rate driven by the laser pulse width and cooling rate driven by the thermal conductivity of the film ultimately determines the range of temperatures within the film particularly the lower temperature. When the “laser-off” period is longer than the cooling period, the sample reaches a lower temperature. When the “laser-off” period is shorter than the cooling period, the sample cools less. This is also observed in the pyroelectric current waveform. Since the pyroelectric current is the direct result of the change in temperature, the magnitude of the current will be proportional to the change in temperature. As this range decreases for the cooling part of the cycle as the repetition rate increases, the resulting negative portion of the pyroelectric current will also decrease. At repetition rates where the cooling rate is competitive with the time between pulses, the change in temperature is large and the pyroelectric current is also larger. As the range of temperature changes decreases, the sample develops a steady-state temperature closer to the maximum temperature. For our samples, when excited with repetition rates faster than 2 Hz, the polarity of the bulk of the waveform is positive current, however, a small negative current is also observed. Our material demonstrates the characteristic of symmetric alternating current signature of pyroelectric current for 0.1 Hz repetition rates or slower.

Figure 8c is a plot of the pyroelectric current density for each of the heating frequencies listed above. The current density is calculated based on the positive portion of the waveforms presented in Fig. 7. The mean current density was 23.7 $\pm$ 1.2 mA/m^2^. Since the current density has been determined from only the positive-going current, the maximum density may be larger. The pyroelectric coefficient was calculated for positive pyroelectric current density at 0.1 Hz heating frequency using Eq. 1 and found to be 3425 μC/m^2^K.

To date, few ferroelectric materials are studied for their pyroelectric measurement and reported their current densities. Bhatia et al*.*^11^ has reported the current density varies from 10^–5^ A/m^2^ to 10^3^ A/m^2^ over the range 10^–2^ to 10^6^ K/s on PbZr_0.2_Ti_0.8_O_3_ thin film. They have observed the current density of 10^–4^, 0.1, and 10^3^ A/m^2^ with temperature oscillation amplitude of 3, 1, and 4.5 K over the heating frequency 0.02, 100, and 10^5^ Hz, respectively. Similarly, Biancoli et al.^40^ has demonstrated a pyroelectric current density of 10 pA/cm^2^ and 3.5 pA/cm^2^ on Ba_0.6_Sr_0.4_TiO_3_ ceramic and BaTiO_3_ single crystal, respectively, with the temperature fluctuation of 1 K and heating frequency 0.02 Hz. Recently, You et al.^41^ demonstrated a pyroelectric current density of 12.5 μA/m^2^ with a temperature oscillation of 6 K on a PVDF polymer at a heating frequency of 0.125 Hz. However, the BCT/BZT thin films, reported here, show relaxor-ferroelectric nature exhibit excellent pyroelectric performance compared to similar lead-free materials reported in the existing literature. The present results show that, in multilayer heterostructures, as the temperature fluctuates, there is abrupt change in polarization due to structural transformation, which in turn, significantly improves the pyroelectric performance. These relaxor-ferroelectric multilayer structures have the potential to be used for future low-grade waste-heat harvesting applications.

## Conclusions

BZT/BCT multilayer nanostructures are successfully grown on SRO coated STO single crystalline substrate using optimized PLD technique. The (*l00*) reflection peaks from STO substrate, SRO, and multilayer structure indicate a crystalline, phase pure, and highly c-axis orientated of these nanostructures. The AFM images, recorded in tapping mode, exhibit a very smooth surface indicating good quality growth. These BZT/BCT multilayer heterostructures demonstrate excellent pyroelectric performance compared to the lead-based materials reported in the existing literature. The above-mentioned results show that these relaxor-ferroelectric multilayer heterostructures demonstrate excellent pyroelectric energy density of 10,970 kJ/m^3^ per cycle and a current density of ~ 25 mA/m^2^ and the corresponding pyroelectric coefficient of 3425 μC/m^2^K with 11.8 °C temperature fluctuation. These pyroelectric results are better than those reported for other lead-free materials and demonstrate a potential to be used in low-grade thermal energy harvesting applications.
